# Finding all *S*-Diophantine quadruples for a fixed set of primes *S*

**DOI:** 10.1007/s00605-021-01605-w

**Published:** 2021-07-19

**Authors:** Volker Ziegler

**Affiliations:** University of Salzburg, Hellbrunnerstrasse 34/I, 5020 Salzburg, Austria

**Keywords:** Baker’s method, *S*-unit equations, *S*-Diophantine tuples, 11D61, 11Y50

## Abstract

Given a finite set of primes *S* and an *m*-tuple $(a_1,\ldots ,a_m)$ of positive, distinct integers we call the *m*-tuple *S*-Diophantine, if for each $1\le i < j\le m$ the quantity $a_ia_j+1$ has prime divisors coming only from the set *S*. For a given set *S* we give a practical algorithm to find all *S*-Diophantine quadruples, provided that $|S|=3$.

## Introduction

Given an irreducible polynomial $f\in {\mathbb {Z}}[X,Y]$ and a set *A* of positive integers we consider the product

$$\begin{aligned} \varPi =\prod _{\begin{array}{c} a,b\in A \\ a\ne b \end{array}} f(a,b). \end{aligned}$$

It is an interesting question what is the largest prime divisor $P(\varPi )$ of $\varPi$ or alternatively what is the number of prime divisors $\omega (\varPi )$ of $\varPi$ in terms of the size of |*A*|. In case of $f(x,y)=x+y$ this question was intensively studied by several authors starting with Erdős and Turán [4] who established that in this case $\omega (\varPi )\gg \log |A|$. It was shown by Erdős, Stewart and Tijdeman [3] that this is essentially best possible.

In the case of $f(x,y)=xy+1$ the investigation was started by Sárközy [8]. It was shown by Győry et.al. that $\omega (\varPi )\gg \log |A|$. However it seems that this lower bound is far from being best possible and the situation is not so clear as in the case $f(x,y)=x+y$.

One recent approach to this problem was picked up by Szalay and Ziegler [11] who studied so-called *S*-Diophantine *m*-tuples. Let *S* be a finite set of primes and $(a_1,\ldots ,a_m)$ a *m*-tuple of positive, distinct integers with $a_1<\dots <a_m$. Then we call $(a_1,\ldots ,a_m)$ *S*-Diophantine, if for each $1\le i < j\le m$ the quantity $a_ia_j+1$ has prime divisors coming only from the set *S*. In other words if $A=\{a_1,\ldots ,a_m\}$ and $\varPi$ has only prime divisors coming form *S*, then $(a_1,\ldots ,a_m)$ is called a *S*-Diophantine *m*-tuple. Szalay and Ziegler [11] conjectured that if $|S|=2$, then no *S*-Diophantine quadruple exists. This conjecture has been confirmed for several special cases by Luca, Szalay and Ziegler [5, 11–13, 16]. Even a rather efficient algorithm has been described by Szalay and Ziegler [13] that finds for a given set $S=\{p,q\}$ of two primes all *S*-Diophantine quadruples, if there exist any. However, the algorithm is strictly limited to the case that $|S|=2$ and does not work (nor any slightly modified version of the algorithm) for $|S|\ge 3$.

In the present paper we establish an algorithm that finds for any set $S=\{p,q,r\}$ of three primes all *S*-Diophantine quadruples. Unfortunately the algorithm is not so efficient as its counter part for $|S|=2$. However, when implemented in Sage [14] it takes on a usual PC (Intel i7-8700) – using a single core – about six and a half hours to verify:

### Theorem 1

There is no $\{2,3,5\}$-Diophantine quadruple.

However the case $S=\{2,3,5\}$ is in some sense the slowest instance for small primes *p*, *q* and *r*. Thus within four days of runtime we could establish the following result:

### Theorem 2

Let $S=\{p,q,r\}$ with $2\le p<q<r\le 100$. Then all *S*-Diophantine quadruples are listed in Table 1.

**Table 1:** *S*-Diophantine quadruples

*S*	Quadruples
$$\{2,3,11\}$$	(1, 3, 5, 7)
$$\{2, 3, 29\}$$	(1, 5, 7, 23)
$$\{2, 11, 37\}$$	(1, 3, 7, 21)

Thus Theorem 2 raises the following problem:

### Problem 1

Are the *S*-Diophantine quadruples listed in Table 1 all *S*-Diophantine quadruples with $|S|=3$?

Although we present the method only in the case that $|S|=3$ obvious modifications to the algorithm would provide an algorithm that would work for any set *S* of primes with $|S|\ge 3$. However, already in the case that $|S|=4$ the algorithm would take instead of several hours several months of computation time. Thus a systematic search for *S*-Diophantine quadruples in the case $|S|=4$ seems to be not feasible. Therefore we refer from discussing the case $|S|>3$ in this paper.

In the next section we remind some of the auxiliary results we need to deal with *S*-Diophantine quadruples. In Sect. 3 we establish several results that will allow us to find small upper bounds for the relevant unknowns and will also allow us to apply LLL-reduction to reduce these bounds. The LLL-reduction will be discussed in Sect. 4. In Sect. 5 we will give details how to apply the LLL-reduction method to find upper bounds small enough to enumerate all possible *S*-Diophantine quadruples for a given set $S=\{p,q,r\}$ of three primes.

## Auxiliary results

Assume that (*a*, *b*, *c*, *d*) is a *S*-Diophantine quadruple, with $S=\{p,q,r\}$. In particular, we assume that $a<b<c<d$. Moreover, we will assume that $p<q<r$. Let us write

$$\begin{aligned} ab+1=s_1&=p^{\alpha _1}q^{\beta _1}r^{\gamma _1}&bc+1=s_4&=p^{\alpha _4}q^{\beta _4}r^{\gamma _4} \\ ac+1=s_2&=p^{\alpha _2}q^{\beta _2}r^{\gamma _2}&bd+1=s_5&=p^{\alpha _5}q^{\beta _5}r^{\gamma _5} \\ ad+1=s_3&=p^{\alpha _3}q^{\beta _3}r^{\gamma _3}&cd+1=s_6&=p^{\alpha _6}q^{\beta _6}r^{\gamma _6}. \end{aligned}$$

The almost trivial observation that

$$\begin{aligned} ab \cdot cd=(s_1-1)(s_6-1)=(s_2-1)(s_5-1)=ac\cdot bd \end{aligned}$$

yields the (non-linear) *S*-unit equation

$$\begin{aligned} s_1s_6-s_1-s_6=s_2s_5-s_2-s_5. \end{aligned}$$

Similar computations lead us to the following system of *S*-unit equations

1 $$\begin{aligned} \begin{aligned} s_1s_6-s_1-s_6&=s_2s_5-s_2-s_5,\\ s_1s_6-s_1-s_6&=s_3s_4-s_3-s_4,\\ s_2s_5-s_2-s_5&=s_3s_4-s_3-s_4. \end{aligned} \end{aligned}$$

Let us call two indices $i,j\in \{1,\ldots ,6\}$ complementary, if $i+j=7$. Note that if *i* and *j* are complementary, then we have $(s_i-1)(s_j-1)=abcd$. With these notations at hand the following lemmas can be proved (e.g. see [11]) by elementary means.

### Lemma 1

The smallest two members of each of the sets

$$\begin{aligned} \{\alpha _1,\alpha _2,\alpha _5,\alpha _6\},\quad \{\alpha _1,\alpha _3,\alpha _4,\alpha _6\},\quad \{\alpha _2,\alpha _3,\alpha _4,\alpha _5\} \end{aligned}$$

are equal. Similar statements also hold for the $\beta$’s and $\gamma$’s

### Proof

This follows by *p*-adic considerations of the system of equations (1) (cf. [11, Proposition 1]). $\square$

### Lemma 2

Assume that (*a*, *b*, *c*, *d*) is a *S*-Diophantine quadruple. Then we have

$$\begin{aligned} a&|\gcd \left( \frac{s_2-s_1}{\gcd (s_2,s_1)},\frac{s_3-s_1}{\gcd (s_3,s_1)},\frac{ s_3-s_2}{\gcd (s_3,s_2)}\right) ,\\ b&|\gcd \left( \frac{s_4-s_1}{\gcd (s_4,s_1)},\frac{s_5-s_1}{\gcd (s_5,s_1)},\frac{ s_5-s_4}{\gcd (s_5,s_4)}\right) ,\\ c&|\gcd \left( \frac{s_4-s_2}{\gcd (s_4,s_2)},\frac{s_6-s_2}{\gcd (s_6,s_2)},\frac{ s_6-s_4}{\gcd (s_6,s_4)}\right) ,\\ d&|\gcd \left( \frac{s_5-s_3}{\gcd (s_5,s_3)},\frac{s_6-s_3}{\gcd (s_6,s_3)},\frac{ s_6-s_5}{\gcd (s_6,s_5)}\right) . \end{aligned}$$

### Proof

A proof can be found in [11, Lemma 3]. $\square$

### Lemma 3

Let (*a*, *b*, *c*) be a *S*-Diophantine triple with

$$\begin{aligned} ab+1&=s_1=p^{\alpha _1}q^{\beta _1}r^{\gamma _1}\\ ac+1&=s_2=p^{\alpha _2}q^{\beta _2}r^{\gamma _2}\\ bc+1&=s_4=p^{\alpha _4}q^{\beta _4}r^{\gamma _4}. \end{aligned}$$

Then we have:

- If $$p=2$$, then $$\min \{\alpha _1,\alpha _2,\alpha _4\}\le 1$$.
- If $$p\equiv 3\mod 4$$, then $$\min \{\alpha _1,\alpha _2,\alpha _4\}=0$$.
- If $$q\equiv 3\mod 4$$, then $$\min \{\beta _1,\beta _2,\beta _4\}=0$$.
- If $$r\equiv 3\mod 4$$, then $$\min \{\gamma _1,\gamma _2,\gamma _4\}=0$$.

### Proof

A proof can be found in [12, Lemma 2.2] except for the case that $p=2$. However, in the case that $p=2$ we slightly change the proof. That is we assume that $\min \{\alpha _1,\alpha _2,\alpha _4\}\ge 2$ and obtain

$$\begin{aligned} a^2b^2c^2=(s_1-1)(s_2-1)(s_3-1)\equiv -1\equiv 3 \pmod 4 \end{aligned}$$

which is an obvious contradiction. $\square$

In order to obtain a first upper bound in the next section we will apply results on lower bounds for linear forms in (complex and *p*-adic) logarithms. Therefore let $\eta \ne 0$ be an algebraic number of degree $\delta$ and let

$$\begin{aligned} a(X-\eta _1)\cdots (X-\eta _\delta ) \in {\mathbb {Z}}[X] \end{aligned}$$

be the minimal polynomial of $\eta$. Then the absolute logarithmic Weil height is defined by

$$\begin{aligned} h(\eta )=\frac{1}{\delta }\left( \log |a|+\sum _{i=1}^\delta \max \{0,\log |\eta _i|\}\right) . \end{aligned}$$

We will mainly need the notation of height for rational numbers. Therefore let us remark that if $p/q \in {\mathbb {Q}}$ with *p*, *q* integers such that $\gcd (p,q)=1$, then $h(p/q)=\max \{\log |p|,\log |q|\}$. With this basic notation we have the following result on lower bounds for linear forms in (complex) logarithms due to Matveev [6].

### Lemma 4

Denote by $\eta _1,\ldots ,\eta _n$ algebraic numbers, nor 0 neither 1, by $\log \eta _1$, $\ldots$, $\log \eta _n$ determinations of their logarithms, by *D* the degree over ${\mathbb {Q}}$ of the number field $K = {\mathbb {Q}}(\eta _1,\ldots ,\eta _n)$, and by $b_1,\ldots ,b_n$ rational integers. Furthermore let $\kappa =1$ if *K* is real and $\kappa =2$ otherwise. Choose

$$\begin{aligned} A_i\ge \max \{D h(\eta _i),|\log \alpha _i|\} \quad (1\le i\le n) \end{aligned}$$

and

$$\begin{aligned} E=\max \{1,\max \{ |b_j| A_j /A_n: 1\le j \le n \}\}. \end{aligned}$$

Assume that $b_n\ne 0$ and $\log \eta _1,\ldots ,\log \eta _n$ are linearly independent over ${\mathbb {Z}}$. Then

$$\begin{aligned}\log |b_1\log \eta _1+\cdots +b_n \log \eta _n|\ge -C(n)C_0 W_0 D^2 \varOmega ,\end{aligned}$$

with

$$\begin{aligned}&\varOmega =A_1\cdots A_n, \\&C(n)=C(n,\kappa )= \frac{16}{n! \kappa } e^n (2n +1+2 \kappa )(n+2) (4(n+1))^{n+1} \left( \frac{1}{2} en\right) ^{\kappa }, \\&C_0= \log \left( e^{4.4n+7}n^{5.5}D^2 \log (eD)\right) , \quad W_0=\log (1.5eED \log (eD)). \end{aligned}$$

In our case of main interest where $|S|=3$ we can apply results on linear forms in two *p*-adic logarithms. For a prime *p* we denote by ${\mathbb {Q}}_p$ the field of *p*-adic numbers with the standard *p*-adic valuation $\nu _p$. As above let $\eta _1,\eta _2$ be algebraic numbers over ${\mathbb {Q}}$ and we regard them as elements of the field $K_p={\mathbb {Q}}_p(\eta _1,\eta _2)$. In our case $\eta _1$ and $\eta _2$ will be rational integers, thus $K_p={\mathbb {Q}}_p$. Similar as in the case of Matveev’s theorem above we have to use a modified height. In particular, we write

$$\begin{aligned} h'(\eta _i) \ge \max \left\{ h(\eta _i),\log p \right\} , \ (i=1,2). \end{aligned}$$

With these notations at hand, let us state the result due to Bugeaud and Laurent [1, Corollary 1]):

### Lemma 5

Let $b_1,b_2$ be positive integers and suppose that $\eta _1$ and $\eta _2$ are multiplicatively independent algebraic numbers such that $\nu _p(\eta _1)=\nu _p(\eta _2)=0$. Put

$$\begin{aligned} E':=\frac{b_1}{h'(\eta _2)}+\frac{b_2}{h'(\eta _1)}. \end{aligned}$$

and

$$\begin{aligned} E:=\max \left\{ \log E'+\log \log p+0.4,10,10\log p\right\} . \end{aligned}$$

Then we have

$$\begin{aligned} \nu _p(\eta _1^{b_1}\eta _2^{b_2}-1)\le \frac{24 p}{(\log p)^4}E^2h'(\eta _1)h'(\eta _2). \end{aligned}$$

The next lemma is an elementary, but rather useful result due to Pethő and de Weger [7]. For a proof of Lemma 6 we refer to [9, Appendix B].

### Lemma 6

Let $u,v \ge 0, h \ge 1$ and $x \in {\mathbb {R}}$ be the largest solution of $x=u+v(\log {x})^h$. Then

$$\begin{aligned} x<\max \{2^h(u^{1/h}+v^{1/h}\log (h^hv))^h, 2^h(u^{1/h}+2e^2)^h\}. \end{aligned}$$

## A first upper bound

As already mentioned in the previous section we consider the case that $|S|=3$, i.e. we have $S=\{p,q,r\}$ with $P=\max \{p,q,r\}$ and write

$$\begin{aligned} ab+1=s_1&=p^{\alpha _1}q^{\beta _1}r^{\gamma _1}&bc+1=s_4&=p^{\alpha _4}q^{\beta _4}r^{\gamma _4} \\ ac+1=s_2&=p^{\alpha _2}q^{\beta _2}r^{\gamma _2}&bd+1=s_5&=p^{\alpha _5}q^{\beta _5}r^{\gamma _5} \\ ad+1=s_3&=p^{\alpha _3}q^{\beta _3}r^{\gamma _3}&cd+1=s_6&=p^{\alpha _6}q^{\beta _6}r^{\gamma _6}. \end{aligned}$$

Let us write

$$\begin{aligned} A=\max _{i=1,\ldots ,6}\{\alpha _i\},\quad B=\max _{i=1,\ldots ,6}\{\beta _i\}, \quad C=\max _{i=1,\ldots ,6}\{\gamma _i\} \end{aligned}$$

and $$M=\max \{A,B,C\}$$.

The main result of this section is the following proposition:

### Proposition 1

If there exists an *S*-Diophantine quadruple, then

$$\begin{aligned} M< 3.62 \cdot 10^{23} (\log P)^5 \log ( 3.62\cdot 10^{23} (\log P)^5)^2. \end{aligned}$$

In the case that $S=\{2,3,5\}$ we have the upper bound

$$\begin{aligned} M<1.26\cdot 10^{28} \end{aligned}$$

and in the case that $S=\{p,q,r\}$ with $p,q,r<100$ we have the upper bound

$$\begin{aligned} M<2.88 \cdot 10^{30}. \end{aligned}$$

First, we prove an upper bound for $\alpha _i,\beta _i$ and $\gamma _i$ with $i=1,2,4$.

### Lemma 7

We have

$$\begin{aligned} \log s_1,\log s_2\le 2.38 \cdot 10^{10} \log p \log q \log r \log (2M) \end{aligned}$$

and

$$\begin{aligned} \log s_4\le 4.76 \cdot 10^{10} \log p \log q \log r \log (2M). \end{aligned}$$

In particular, we have

$$\begin{aligned} \alpha _1,\alpha _2&\le 2.38 \cdot 10^{10} \log q \log r \log (2M),\\ \beta _1,\beta _2&\le 2.38 \cdot 10^{10} \log p \log r \log (2M),\\ \gamma _1,\gamma _2&\le 2.38 \cdot 10^{10} \log p \log q \log (2M), \end{aligned}$$

and

$$\begin{aligned} \alpha _4&\le 4.76 \cdot 10^{10} \log q \log r \log (2M),\\ \beta _4&\le 4.76 \cdot 10^{10} \log p \log r \log (2M),\\ \gamma _4&\le 4.76 \cdot 10^{10} \log p \log q \log (2M). \end{aligned}$$

### Proof

Following Stewart and Tijdeman [10] we consider the quantity

2 $$\begin{aligned} T_1=p^{A'}q^{B'}r^{C'}=\frac{(ab+1)(cd+1)}{(ac+1)(bd+1)}=1+\frac{cd+ab-ac-bd}{(ac+1)(bd+1)}=1+\frac{\theta }{ab+1}\nonumber \\ \end{aligned}$$

with $|\theta |< 1$, $A'=\alpha _1+\alpha _6-\alpha _2-\alpha _5$, $B'=\beta _1+\beta _6-\beta _2-\beta _5$ and $C'=\gamma _1+\gamma _6-\gamma _2-\gamma _5$. Note that we have $|A'|\le 2A$, $|B'|\le 2B$ and $|C'|\le 2C$. Let us note that $\log (1+x)\le 2x$ provided that $|x|\le 1/2$. Since $ab+1\ge 2$ we obtain by taking logarithms

3 $$\begin{aligned} |\varLambda _1|=|A' \log p+B'\log q+C'\log r|<\frac{2}{p^{\alpha _1}q^{\beta _1}r^{\gamma _1}}=\frac{2}{s_1} \end{aligned}$$

We apply Matveev’s theorem (Lemma 4) with

$$\begin{aligned} D=1, \quad n=3, \quad \kappa =1, \quad b_1=A',\quad b_2=B',\quad b_3=C', \\ \eta _1=p, \quad \eta _2=q, \quad \eta _3=r, \quad A_1=\log p, \quad A_2=\log q, \quad A_3=\log r. \end{aligned}$$

Therefore we have $E\le \max \{A',B',C'\}\le 2M$ provided $M>2$ which we clearly may assume. Thus we obtain

$$\begin{aligned} \log s_1 -\log 2 < 2.375 \cdot 10^{10} \log p \log q \log r \log (2M), \end{aligned}$$

which yields the upper bound for $\log s_1$. Since

$$\begin{aligned} \log s_1=\alpha _1\log p+\beta _1 \log q+\gamma _1\log r \end{aligned}$$

this also yields the upper bounds for $\alpha _1,\beta _1$ and $\gamma _1$.

If we consider instead of $T_1$ the quantity

4 $$\begin{aligned} T_2=p^{A''}q^{B''}r^{C''}=\frac{(ac+1)(bd+1)}{(bc+1)(ad+1)} =1+\frac{bd+ac-bc-ad}{(bc+1)(ad+1)}=1+\frac{\theta }{ac+1}\nonumber \\ \end{aligned}$$

with $|\theta |< 1$, $A''=\alpha _2+\alpha _5-\alpha _3-\alpha _4$, $B''=\beta _2+\beta _5-\beta _3-\beta _4$ and $C''=\gamma _2+\gamma _5-\gamma _3-\gamma _4$. We end up with the linear form

5 $$\begin{aligned} |\varLambda _2|=|A'' \log p+B''\log q+C''\log r|<\frac{2}{p^{\alpha _2}q^{\beta _2}r^{\gamma _2}} \end{aligned}$$

and again an application of Matveev’s result yields the same upper bound for $\log s_2$ and also the same upper bounds for $\alpha _2,\beta _2$ and $\gamma _2$.

Finally let us note that

$$\begin{aligned} s_4= bc+1<(ab+1)(ac+1)=s_1s_2, \end{aligned}$$

which yields after some easy computations the stated upper bounds for $\log s_4$, $\alpha _4$, $\beta _4$ and $\gamma _4$. $\square$

Let us denote by $M_0$ and $M_4$ upper bounds for $\max \{\log s_1,\log s_2\}$ and $\log s_4$ respectively. Then the previous lemma provides upper bounds for $M_0$ and $M_4$.

### Lemma 8

We have $$M\le 3.79 \cdot 10^{12} M_0 (\log P)^2 \log M$$.

### Proof

Following again the arguments of Stewart and Tijdeman [10] we consider the following inequality

$$\begin{aligned} 0\le \frac{b}{a} p^{\alpha _3-\alpha _5}q^{\beta _3-\beta _5}r^{\gamma _3-\gamma _5}-1=\frac{b}{a} \cdot \frac{ad+1}{bd+1}-1=\frac{b-a}{a(bd+1)}\le \frac{1}{d} \end{aligned}$$

which implies

6 $$\begin{aligned} \varLambda _3:=\left| \log \frac{b}{a}+A' \log p + B'\log q +C' \log r\right| \le \frac{2}{d}, \end{aligned}$$

where $A'=\alpha _3-\alpha _5$, $B'=\beta _3-\beta _5$ and $C'=\gamma _3-\gamma _5$. We apply Matveev’s result with

$$\begin{aligned} D=1, \quad n=4, \quad \kappa =1,\quad b_1=1, \quad b_2=A',\quad b_3=B',\quad b_4=C', \\ \eta _1=\frac{a}{b}, \quad \eta _1=p, \quad \eta _2=q, \quad \eta _3=r,\\ A_1=\log \left( \max \{a,b\}\right) , \quad A_2=\log p, \quad A_3=\log q, \quad A_4=\log r. \end{aligned}$$

Since

$$\begin{aligned} b<ab+1=\exp (\alpha _1 \log p+\beta _1 \log q+\gamma _1\log r)\le \exp (M_0) \end{aligned}$$

and $\max \{A',B',C'\}\le M$ we get

$$\begin{aligned} 1.893\cdot 10^{12} M_0 \log p \log q \log r \log M >\log d-\log 2. \end{aligned}$$

On the other hand we have

$$\begin{aligned} d^2>dc+1&\ge \exp \left( \max \{A\log p, B \log q, C \log r\}\right) \\&\ge \exp (M\min \{\log p,\log q,\log r\}). \end{aligned}$$

Combining these two inequalities yields the content of the lemma. $\square$

Now the proof of Proposition 1 is a combination of Lemmas 7 and 8.

### Proof of Proposition 1

By inserting the bound for $M_0$ obtained by Lemma 7 into the bound provided by Lemma 8 we obtain the following inequality

$$\begin{aligned} M< 9.03 \cdot 10^{22} (\log P)^5 (\log 2M)^2. \end{aligned}$$

An application of Lemma 6 yields

$$\begin{aligned} M< 3.62 \cdot 10^{23} (\log P)^5 \log ( 3.62\cdot 10^{23} (\log P)^5)^2. \end{aligned}$$

If we put $P=5$ and $P=100$ we obtain the other bounds stated in the proposition. $\square$

Although we have an upper bound for *M* in Proposition 1 this upper bound provides only rather huge bounds for all the exponents. Moreover, the linear form of logarithms (6) is not suitable for applying standard reduction schemes like LLL-reduction. In particular, there are far too many possible pairs (*a*, *b*) left to apply for each possible pair the LLL-reduction to the linear form of logarithms (6) to get in each case a lower bound. Therefore we give a more detailed account on how to find smaller upper bounds for all the exponents.

First, we will prove that all exponents with one possible exception can be bounded in terms of $M_0$ and $M_4$. In particular, Proposition 2 will be useful in the bound reduction process which we will describe in Sect. 5. Before we can state and prove Proposition 2 we prove another helpful lemma:

### Lemma 9

There exists a permutation $\sigma$ of $\{1,\ldots ,6\}$ such that

$$\begin{aligned} \alpha _{\sigma (1)}\le \alpha _{\sigma (2)}\le \alpha _{\sigma (3)}\le \alpha _{\sigma (4)}\le \alpha _{\sigma (5)}\le \alpha _{\sigma (6)}. \end{aligned}$$

and such that one of the following two relations holds:

- $\alpha _{\sigma (1)}=\alpha _{\sigma (2)}=\alpha _{\sigma (3)}\le \alpha _{\sigma (4)} \le \alpha _{\sigma (5)}\le \alpha _{\sigma (6)}$ and no two indices out of $\sigma (1)$, $\sigma (2)$ and $\sigma (3)$ are complementary,
- $\alpha _{\sigma (1)}=\alpha _{\sigma (2)}\le \alpha _{\sigma (3)}=\alpha _{\sigma (4)} \le \alpha _{\sigma (5)}\le \alpha _{\sigma (6)}$ and $\sigma (1)$ and $\sigma (2)$ are complementary.

### Proof

Let $\sigma$ be any permutation of $\{1,\ldots ,6\}$ such that

$$\begin{aligned} \alpha _{\sigma (1)}\le \alpha _{\sigma (2)}\le \alpha _{\sigma (3)}\le \alpha _{\sigma (4)}\le \alpha _{\sigma (5)}\le \alpha _{\sigma (6)}. \end{aligned}$$

Obviously such a permutation exists.

Since Lemma 1 we know that $\alpha _{\sigma (1)}=\alpha _{\sigma (2)}$. Assume that $\sigma (1)$ and $\sigma (2)$ are not complementary. Then there is exactly one *S*-unit equation out of the system (1) such that this unit equation contains the index $\sigma (1)$ but not $\sigma (2)$. Since $\alpha _{\sigma (1)}$ is minimal it is equal to one exponent of this unit equation, i.e. $\alpha _{\sigma (1)}=\alpha _{\sigma (2)}= \alpha _{\sigma (3)}$.

If $\sigma (1)$ and $\sigma (3)$ are complementary then there is exactly one *S*-unit equation out of the system (1) that does not contain the indices $\sigma (1)$ and $\sigma (3)$ but contains the index $\sigma (2)$. But since $\alpha _{\sigma (2)}$ is minimal it is equal to $\alpha _{\sigma (4)}$ and we have $\alpha _{\sigma (1)}=\alpha _{\sigma (2)}=\alpha _{\sigma (3)}= \alpha _{\sigma (4)}$. By exchanging the values of $\sigma (2)$ and $\sigma (3)$ the numbers $\sigma (1)$ and $\sigma (2)$ are complementary and we have found a permutation that satisfies the second case described in the lemma. A similar argument holds if $\sigma (2)$ and $\sigma (3)$ are complementary. Thus we have proved: If $\sigma (1)$ and $\sigma (2)$ are not complementary, then either the first case holds or the second case holds after rearranging the order of the $\alpha$’s, i.e. we have found a suitable permutation $\sigma$.

Therefore let us assume that $\sigma (1)$ and $\sigma (2)$ are complementary. Then there is a unique *S*-unit equation out of the system (1) such that this unit equation does not contain the indices $\sigma (1)$ and $\sigma (2)$. Thus by Lemma 1 we obtain that $\alpha _{\sigma (3)}= \alpha _{\sigma (4)}$ and we are in the second case of the lemma. $\square$

Similar as in the case of the exponents of *p* we let $\tau$ and $\rho$ be permutations of $\{1,\ldots ,6\}$ such that

$$\begin{aligned} \beta _{\tau (1)}\le \beta _{\tau (2)}\le \beta _{\tau (3)}\le \beta _{\tau (4)}\le \beta _{\tau (5)}\le \beta _{\tau (6)} \end{aligned}$$

and

$$\begin{aligned} \gamma _{\rho (1)}\le \gamma _{\rho (2)}\le \gamma _{\rho (3)}\le \gamma _{\rho (4)}\le \gamma _{\rho (5)}\le \gamma _{\rho (6)} \end{aligned}$$

respectively. By exchanging the roles of the $\alpha$’s, $\beta$’s and $\gamma$’s we can show that permutations $\tau$ and $\rho$ with analogous properties as stated in Lemma 9 exist. Let us fix the permutations $\sigma , \rho$ and $\tau$. We are now in a position to state the next proposition that will prove to be useful in reducing the huge upper bounds we get from Proposition 1.

### Proposition 2

Assume that $\log s_1,\log s_2 \le M_0$ and that $\log s_4\le M_4$. Moreover, let

$$\begin{aligned} B_p&=\log p \max _{\begin{array}{c} |\beta | \le M_4/\log q \\ |\gamma | \le M_4/\log r \end{array}}\left\{ \nu _p(q^\beta r^\gamma -1)\right\} ,\\ B_q&=\log q \max _{\begin{array}{c} |\alpha | \le M_4/\log p \\ |\gamma | \le M_4/\log r \end{array}}\left\{ \nu _q(p^\alpha r^\gamma -1)\right\} ,\\ B_r&=\log r \max _{\begin{array}{c} |\alpha | \le M_4/\log p \\ |\beta | \le M_4/\log q \end{array}}\left\{ \nu _r(p^\alpha q^\beta -1)\right\} \end{aligned}$$

and ${\mathcal {B}}=\max \{B_p,B_q,B_r\}$. Then we have

$$\begin{aligned} \alpha _{\sigma (5)}\le \frac{\max \{M_0+B_p,M_4\}}{\log p},\qquad \beta _{\tau (5)}\le \frac{\max \{M_0+B_q,M_4\}}{\log q},\\ \gamma _{\rho (5)}\le \frac{\max \{M_0+B_r,M_4\}}{\log r}. \end{aligned}$$

In particular, we have

$$\begin{aligned} \max \{\alpha _{\sigma (5)} \log p ,\beta _{\tau (5)} \log q ,\gamma _{\rho (5)} \log r\} \le \max \{M_0+{\mathcal {B}},M_4\}:=M_5. \end{aligned}$$

### Proof

We give only the details for the proof of the upper bound for $\alpha _{\sigma (5)}$ since the upper bounds for $\beta _{\tau (5)}$ and $\gamma _{\rho (5)}$ can be deduced by the same argument.

First, let us assume that we are in the first case of Lemma 9. Let us assume for the moment that $|\{1,2,4\}\cap \{\sigma (1),\sigma (2),\sigma (3)\}|\le 1$. In this case we conclude that $|\{1,2,4\}\cap \{\sigma (4),\sigma (5),\sigma (6)\}|\ge 2$ and therefore $\sigma (5)\in \{1,2,4\}$ or $\sigma (6)\in \{1,2,4\}$. Thus in any case $\alpha _{\sigma (5)} \le M_4/\log p$.

Now, let us assume that we are again in the first case of Lemma 9 and that $|\{1,2,4\}\cap \{\sigma (1),\sigma (2),\sigma (3)\}|= 2$. We may assume that $\sigma (3) \not \in \{1,2,4\}$. If either $\sigma (5)$ or $\sigma (6)$ are contained in $\{1,2,4\}$, then we easily conclude that $\alpha _{\sigma (5)} \le M_4/\log p$. Thus we may assume that $\sigma (4)\in \{1,2,4\}$. Further, we deduce that $\sigma (3)$ and $\sigma (4)$ are complementary, and without loss of generality we may assume that $\sigma (1)$ and $\sigma (6)$ as well as $\sigma (2)$ and $\sigma (5)$ are complementary. Let us consider the unit equation

$$\begin{aligned} s_{\sigma (6)}s_{\sigma (1)} - s_{\sigma (6)} -s_{\sigma (1)}=s_{\sigma (5)}s_{\sigma (2)} - s_{\sigma (5)} -s_{\sigma (2)}. \end{aligned}$$

Dividing through $p^{\alpha _{\sigma (1)}}$ and collecting the terms not divisible by *p* on one side of the equation we obtain

$$\begin{aligned} q^{\beta _{\sigma (2)}}r^{\gamma _{\sigma (2)}} -q^{\beta _{\sigma (1)}}r^{\gamma _{\sigma (1)}}= p^{\alpha _{\sigma (5)}-\alpha _{\sigma (1)}} \times {\mathcal {N}} \end{aligned}$$

where ${\mathcal {N}}$ is an integer. Thus we deduce that

$$\begin{aligned} \frac{{\mathcal {B}}}{\log p}\ge \frac{B_p}{\log p}\ge \nu _p\left( q^{\beta _{\sigma (2)}-\beta _{\sigma (1)}}r^{\gamma _{\sigma (2)} -\gamma _{\sigma (1)}}-1 \right) \\ \ge \alpha _{\sigma (5)}-\alpha _{\sigma (1)}\ge \alpha _{\sigma (5)}- \frac{M_0}{\log p} \end{aligned}$$

which implies the upper bound for $\alpha _{\sigma (5)}$. Note that by our choice $\sigma (1),\sigma (2)\in \{1,2,4\}$ the exponents satisfy $|\beta _{\sigma (2)}-\beta _{\sigma (1)}|\le \beta _4$ and $|\gamma _{\sigma (2)}-\gamma _{\sigma (1)}|\le \gamma _4$.

Assume again that we are in the first case of Lemma 9 and that $\{1,2,4\}= \{\sigma (1),\sigma (2),\sigma (3)\}$. In this case we can choose $\sigma$ such that $\sigma (1)$ and $\sigma (6)$ as well as $\sigma (2)$ and $\sigma (5)$ are complementary. Now the same line of arguments as in the previous paragraph yields the same upper bound for $\alpha _{\sigma (5)}$.

Now, we assume that we are in the second case of Lemma 9. Since $\sigma (1)$ and $\sigma (2)$ are complementary exactly one of the two is an element of $\{1,2,4\}$. Assume now that $|\{\sigma (3),\sigma (4)\}\cap \{1,2,4\}|\le 1$, then either $\sigma (5)$ or $\sigma (6)$ is contained in $\{1,2,4\}$ and we deduce in any case that $\alpha _{\sigma (5)}\le M_4/\log p$. Therefore we may assume that $\sigma (3),\sigma (4)\in \{1,2,4\}$. However $\sigma (1)$ and $\sigma (2)$ are complementary and since $\sigma (3)$ and $\sigma (4)$ are not we can choose $\sigma$ such that $\sigma (3)$ and $\sigma (5)$ as well as $\sigma (4)$ and $\sigma (6)$ are complementary. By the same reasoning as in the proof of the first case we deduce that

$$\begin{aligned} \frac{{\mathcal {B}}}{\log p}\ge \frac{B_p}{\log p}\ge \nu _p\left( q^{\beta _{\sigma (4)}-\beta _{\sigma (3)}}r^{\gamma _{\sigma (4)}-\gamma _{\sigma (3)}}-1 \right) \ge \alpha _{\sigma (5)}-\alpha _{\sigma (3)}. \end{aligned}$$

Since we are in the second case of Lemma 9 we know that $\alpha _{\sigma (1)}$ and $\alpha _{\sigma (2)}$ are complementary and therefore

$$\begin{aligned} \{\sigma (3),\sigma (4),\sigma (5),\sigma (6)\} \cap \{1,2\}\ne \emptyset \end{aligned}$$

which implies that $\alpha _{\sigma (3)}\le M_0/\log p$ and we obtain the upper bound for $\alpha _{\sigma (5)}$ also in the second case of the proposition. $\square$

The next lemma shows that if *M* is large, then the indices of the large exponents are of a special form.

### Lemma 10

Let us assume that $\alpha _{\sigma (5)}\le M_5/\log p,\beta _{\tau (5)}\le M_5/\log q$ and $\gamma _{\rho (5)}\le M_5/\log r$. Then either $M\le \frac{M_0+3M_5}{\log (\min \{p,q,r\})}$ or $\{\sigma (6),\tau (6),\rho (6)\}=\{3,5,6\}$.

### Proof

Assume that $\{\sigma (6),\tau (6),\rho (6)\}\ne \{3,5,6\}$ and let $*\in \{3,5,6\}$ be such that $*\not \in \{\sigma (6),\tau (6),\rho (6)\}$. We obviously have that

$$\begin{aligned} d<s_*\le p^{\alpha _{\sigma (5)}}q^{\beta _{\tau (5)}}r^{\gamma _{\rho (5)}}\le \exp (3M_5) \end{aligned}$$

and also

$$\begin{aligned} c<s_2=p^{\alpha _2}q^{\beta _2}r^{\gamma _2}\le \exp (M_0). \end{aligned}$$

Therefore we obtain

$$\begin{aligned} \max \{p^{\alpha _{\sigma (6)}},q^{\beta _{\tau (6)}},r^{\gamma _{\rho (6)}}\}\le s_6=cd+1\le \exp (M_0+3M_5). \end{aligned}$$

Now, taking logarithms yields the content of the lemma. $\square$

Let us denote by ${\mathbb {N}}=\{0,1,2,\ldots \}$ the set of the non negative integers. Now, we can provide upper bounds for the largest exponents.

### Proposition 3

Let us assume that $M> \frac{M_0+3M_5}{\log (\min \{p,q,r\})}$, $\sigma (6)$ and *x* are complementary indices and that *y*, *z* is another pair of complementary indices, with $y\in \{1,2,4\}$. If $\alpha _y=\alpha _z<\alpha _x$ (Case I) we put

$$\begin{aligned}&{\mathcal {M}}_{x,y,z}=\left\{ (a_x,b_x,c_x,a_y,b_y,c_y,b_z,c_z)\in {\mathbb {N}}^8\, :\right. \\&1\le p^{a_x}q^{b_x}r^{c_x}, p^{a_y}q^{b_y}r^{c_y}\le \exp (M_4),\; a_y<a_x,\; \text {and}\\&\left. \left( 0\le b_z\le \frac{M_5}{\log q},0\le c_z\le M \; \text {or}\;\; 0\le b_z\le M ,0\le c_z\le \frac{M_5}{\log r} \right) \right\} , \end{aligned}$$

if $\alpha _x=\alpha _z<\alpha _y$ (Case II) we put

$$\begin{aligned}&{\mathcal {M}}_{x,y,z}=\left\{ (a_x,b_x,c_x,a_y,b_y,c_y,b_z,c_z)\in {\mathbb {N}}^8\, :\right. \\&1\le p^{a_x}q^{b_x}r^{c_x}, p^{a_y}q^{b_y}r^{c_y}\le \exp (M_4), \; a_x<a_y,\; \text {and}\\&\left. \left( 0\le b_z\le \frac{M_5}{\log q},0\le c_z\le M \; \text {or}\;\; 0\le b_z\le M ,0\le c_z\le \frac{M_5}{\log r} \right) \right\} , \end{aligned}$$

and if $\alpha _x=\alpha _y$ (Case III) we put

$$\begin{aligned}&{\mathcal {M}}_{x,y,z}=\left\{ (a_x,b_x,c_x,a_y,b_y,c_y,b_z,c_z)\in {\mathbb {N}}^8\, :\right. \\&1\le p^{a_x}q^{b_x}r^{c_x}, p^{a_y}q^{b_y}r^{c_y}\le \exp (M_4), \; a_x=a_y,\; \text {and}\\&\left. \left( 0\le b_z\le \frac{M_5}{\log q},0\le c_z\le M \; \text {or}\;\; 0\le b_z\le M ,0\le c_z\le \frac{M_5}{\log r} \right) \right\} . \end{aligned}$$

We define

$$\begin{aligned} {\mathcal {C}}_p&=\max _{{\mathcal {M}}_{x,y,z}}\left\{ \nu _p\left( \frac{q^{b_z}r^{c_z}\left( p^{a_y}q^{b_y}r^{c_y}-1\right) }{q^{b_y}r^{c_y}-p^{a_x-a_y}q^{b_x}r^{c_x}}-1\right) \right\}&\text {Case I},\\ {\mathcal {C}}_p&=\max _{{\mathcal {M}}_{x,y,z}}\left\{ \nu _p\left( \frac{q^{b_z}r^{c_z}\left( p^{a_y}q^{b_y}r^{c_y}-1\right) }{p^{a_y-a_x}q^{b_y}r^{c_y}-q^{b_x}r^{c_x}}-1\right) \right\}&\text {Case II},\\ {\mathcal {C}}_p&=\max _{{\mathcal {M}}_{x,y,z}}\left\{ \nu _p\left( \frac{q^{b_z}r^{c_z}\left( p^{a_y}q^{b_y}r^{c_y}-1\right) _p}{ \left( q^{b_y}r^{c_y}-q^{b_x}r^{c_x}\right) _p}-1 \right) \right\}&\text {Case III},\\ \end{aligned}$$

where $(\cdot )_p$ denotes the *p*-free part. Then

$$\begin{aligned} \alpha _{\sigma (6)}\le \left\{ \begin{array}{cl} \frac{M_4}{\log p}+{\mathcal {C}}_p &{} \text {Cases I and II;}\\ \\ \frac{M_4+B_p}{\log p}+{\mathcal {C}}_p &{} \text {Case III.}\end{array} \right. \end{aligned}$$

### Proof

Let us assume that Case I holds. Then we consider the *S*-unit equation

$$\begin{aligned} s_zs_y-s_z-s_y=s_{\sigma (6)}s_x -s_{\sigma (6)}-s_x \end{aligned}$$

which turns into

$$\begin{aligned} \frac{s_z(s_y-1)}{s_y-s_x} -1=\frac{s_{\sigma (6)} (s_x-1)}{s_y-s_x}. \end{aligned}$$

First, we note that $\nu _p(s_y-s_x)=\alpha _y=\alpha _z$ and since $\alpha _x>0$ we have

7 $$\begin{aligned} \nu _p\left( \frac{s_{\sigma (6)} (s_x-1)}{s_y-s_x}\right) =\alpha _{\sigma (6)}-\alpha _y. \end{aligned}$$

Note that with these notations and assumptions we have after canceling a common factor $p^{\alpha _y}=p^{\alpha _z}$ the equation

$$\begin{aligned} \frac{s_z(s_y-1)}{s_y-s_x} -1=\frac{q^{\beta _z}r^{\gamma _z}\left( p^{\alpha _y}q^{\beta _y}r^{\gamma _y} -1\right) }{q^{\beta _y}r^{\gamma _y}-p^{\alpha _x}q^{\beta _x}r^{\gamma _x}}-1 \end{aligned}$$

with non negative exponents $\alpha _x,\alpha _y,\beta _x,\beta _y,\gamma _x$ and $\gamma _y$ satisfying $\alpha _y<\alpha _x$ and

$$\begin{aligned} 0\le \alpha _x\log p+\beta _x\log q+\gamma _x\log r,\alpha _y\log p+\beta _y\log q+\gamma _y\log r \le M_4. \end{aligned}$$

Moreover due to Lemma 10 we have either $0\le \beta _z\le \frac{M_5}{\log q}$ and $0\le \gamma _z\le M$ or $0\le \beta _z\le M$ and $0\le \gamma _z\le \frac{M_5}{\log r}$. That is $(\alpha _x,\beta _x,\gamma _x,\alpha _y,\beta _y,\gamma _y,\beta _z,\gamma _z)\in {\mathcal {M}}_{x,y,z}$. Thus we conclude that

$$\begin{aligned} {\mathcal {C}}_p\ge \nu _p \left( \frac{s_z(s_y-1)}{s_y-s_x} -1\right) =\nu _p\left( \frac{s_{\sigma (6)} (s_x-1)}{s_y-s_x}\right) =\alpha _{\sigma (6)}-\alpha _y. \end{aligned}$$

Which implies in view of $\alpha _y\le M_4/\log p$ and (7) the upper bound for $\alpha _{\sigma (6)}$.

Almost the same arguments apply in the case that $\alpha _x=\alpha _z<\alpha _y$ holds, i.e. that Case II holds. We only have to exchange the roles of *x* and *y*.

In Case III, that is we have $\alpha _x=\alpha _y$, the proof runs along similar arguments. That is we consider the equation

$$\begin{aligned} \frac{s_z(s_y-1)}{s_y-s_x} -1=\frac{s_{\sigma (6)} (s_x-1)}{s_y-s_x}. \end{aligned}$$

However this time we note that

$$\begin{aligned} \nu _p(s_y-s_x)= \alpha _x+\nu _p(q^{\beta _y-\beta _x}r^{\gamma _y-\gamma _x}-1) \le \frac{M_4+B_p}{\log p}. \end{aligned}$$

This implies that

$$\begin{aligned} \alpha _{\sigma (6)}\le \nu _p\left( \frac{s_z(s_y-1)}{s_y-s_x} -1\right) +\nu _p(s_y-s_x), \end{aligned}$$

and therefore the upper bound for $\alpha _{\sigma (6)}$. Finally let us note that a non vanishing factor *p* of $\frac{s_z(s_y-1)}{s_y-s_x}$ would imply that $\nu _p\left( \frac{s_z(s_y-1)}{s_y-s_x} -1\right) \le 0$, thus we may eliminate all possible powers of *p* in the formula for ${\mathcal {C}}_p$, which accounts in the usage of *p*-free parts in the formulas for Case II. $\square$

Let us note that we can prove similar results for $\beta _{\tau (6)}$ and $\gamma _{\rho (6)}$ by exchanging the roles of *p*, *q* and *r* respectively. For the sake of completeness we state the results for $\beta _{\tau (6)}$ and $\gamma _{\rho (6)}$ in the “Appendix” but do not provide a proof since the proofs are identical after an appropriate permutation of *p*, *q* and *r*.

If we have no explicit bounds for $M_4$ and $M_5$ this proposition is hard to apply. However, if we have small, explicit upper bounds for $M_4$ and $M_5$ we can estimate ${\mathcal {C}}_p$ by applying lower bounds for linear forms in two *p*-adic logarithms (for details see Sect. 5) which will yield reasonable small bound for *M*.

## The LLL-reduction

In this section we gather some basic facts on LLL-reduced bases, approximation lattices and their applications to Diophantine problems as they can be found in [9, Chapters 5 and 6].

Let ${\mathcal {L}}\subseteq {\mathbb {R}}^k$ be a *k*-dimensional lattice with LLL-reduced basis $b_1,\ldots ,b_k$ and denote by *B* be the matrix with columns $b_1,\ldots , b_k$. Moreover, we denote by $b^*_1,\ldots ,b^*_k$ the orthogonal basis of ${\mathbb {R}}^k$ which we obtain by applying the Gram-Schmidt process to the basis $b_1,\ldots ,b_k$. In particular, we have that

$$\begin{aligned} b^*_i=b_i-\sum _{j=1}^{i-1}\mu _{i,j}b^*_j, \qquad \mu _{i,j}=\frac{\langle b_i,b_j\rangle }{\langle b_j^*,b_j^*\rangle }. \end{aligned}$$

Further, let us define

$$\begin{aligned} l({\mathcal {L}},y)={\left\{ \begin{array}{ll} \min _{x \in {\mathcal {L}}} \Vert x-y\Vert , &{} y \not \in {\mathcal {L}}\\ \min _{0 \ne x \in {\mathcal {L}}} \Vert y\Vert , &{} y \in {\mathcal {L}}, \end{array}\right. } \end{aligned}$$

where $\Vert \cdot \Vert$ denotes the euclidean norm on ${\mathbb {R}}^k$. It is well known, that by applying the LLL-algorithm it is possible to give in polynomial time a lower bound for $l({\mathcal {L}},y) \ge {{\tilde{c}}}_1$ (see e.g. [9, Section 5.4]):

### Lemma 11

Let $y\in {\mathbb {R}}^k$, $z=B^{-1}y$. Furthermore we define

- If $y\not \in {\mathcal {L}}$ let $i_0$ be the largest index such that $z_{i_0}\ne 0$ and put $\sigma =\{z_{i_0}\}$, where $\{\cdot \}$ denotes the distance to the nearest integer.
- If $y\in {\mathcal {L}}$ we put $\sigma =1$.

Finally let

$$\begin{aligned} {{\tilde{c}}}_2=\max _{1\le j\le k}\left\{ \frac{\Vert b_1\Vert ^2}{\Vert b_j^*\Vert ^2} \right\} . \end{aligned}$$

Then we have

$$\begin{aligned} l({\mathcal {L}},y)^2\ge {{\tilde{c}}}_2^{-1}\sigma ^2 \Vert b_1\Vert ^2={{\tilde{c}}}_1. \end{aligned}$$

In our applications we suppose that we are given real numbers $\eta _0,\eta _1,\ldots ,\eta _k$ linearly independent over ${\mathbb {Q}}$ and two positive constants ${{\tilde{c}}}_3,{{\tilde{c}}}_4$ such that

8 $$\begin{aligned} |\eta _0+x_1\eta _1+\dots +x_k\eta _k| \le {{\tilde{c}}}_3\exp (-{{\tilde{c}}}_4H), \end{aligned}$$

where the integers $x_i$ are bounded by $|x_i| \le X_i$ with $X_i$ given upper bounds for $1 \le i \le k$. We write $X_0=\max _{1 \le i \le s}\{X_i\}$. The basic idea in such a situation, due to de Weger [2], is to approximate the linear form (8) by an approximation lattice. Namely, we consider the lattice ${\mathcal {L}}$ generated by the columns of the matrix

$$\begin{aligned} {\mathcal {A}}=\begin{pmatrix} 1 &{} 0 &{} \dots &{} 0 &{} 0 \\ 0 &{} 1 &{} \dots &{} 0 &{} 0 \\ \vdots &{} \vdots &{} \vdots &{} \vdots &{} \vdots \\ 0 &{} 0 &{} \dots &{} 1 &{} 0 \\ \lfloor {{{\tilde{C}}}\eta _1}\rfloor &{} \lfloor {{{\tilde{C}}}\eta _2}\rfloor &{} \dots &{} \lfloor {{{\tilde{C}}}\eta _{k-1}}\rfloor &{} \lfloor {{{\tilde{C}}}\eta _k}\rfloor \end{pmatrix} \end{aligned}$$

where ${{\tilde{C}}}$ is a large constant usually of the size of about $X_0^k$. Let us assume that we have an LLL-reduced basis $b_1,\ldots ,b_k$ of ${\mathcal {L}}$ and that we have a lower bound $l({\mathcal {L}},y)>{{\tilde{c}}}_1$ with $y=(0,0,\ldots ,-\lfloor {C\eta _0}\rfloor )$. Note that ${{\tilde{c}}}_1$ can be computed by using the results of Lemma 11. Then we have with these notations the following lemma (e.g. see [9, Lemma VI.1]):

### Lemma 12

Assume that $S=\sum _{i=1}^{k-1}X_i^2$ and $T=\frac{1+\sum _{i=1}^k{X_i}}{2}$. If ${{\tilde{c}}}_1^2 \ge T^2+S$, then inequality (8) implies that we have either $x_1=x_2=\dots =x_{k-1}=0$ and $x_k=-\frac{\lfloor {{{\tilde{C}}}\eta _0}\rfloor )}{\lfloor {{{\tilde{C}}}\eta _k}\rfloor )}$ or

9 $$\begin{aligned} H \le \frac{1}{{{\tilde{c}}}_4}\left( \log ({{\tilde{C}}}{{\tilde{c}}}_3)-\log \left( \sqrt{{{\tilde{c}}}_1^2-S}-T\right) \right) . \end{aligned}$$

## Reduction of the bounds

In this section we describe how we can reduce the rather huge bounds for the exponents $\alpha _i,\beta _i,\gamma _i$ for $i=1,\ldots ,6$ for a given set of primes $S=\{p,q,r\}$ and how it is in practice possible to enumerate for the given set *S* all *S*-Diophantine quadruples. We give the full details for the proof of Theorem 1, i.e. the case that $S=\{2,3,5\}$ and will outline the strategy how to find all *S*-Diophantine quadruples for a general set of three primes *S*. The reduction process follows in eight steps:

**Step I** We compute an upper bound for *M* by using Proposition 1. In the case that $S=\{2,3,5\}$ we obtain $M<1.26\cdot 10^{28}$. Note that in the case that $P<100$ which is relevant for the proof of Theorem 2 we obtain the bound $M<2.88\cdot 10^{30}$.

**Step II** We apply the LLL-reduction method explained in Sect. 4 to the linear forms of logarithms (3) and (5) in order to obtain a small bound for $M_0$.

In particular, we apply Lemma 12 to the linear form (3). In the case that $S=\{2,3,5\}$ we put $k=3$ and $\eta _1=\log 5, \eta _2=\log 3$ and $\eta _3=\log 2$. Moreover we have $0\le A',B',C'\le 2M$, i.e. we have to put $X_1=X_2=X_3=2.52\cdot 10^{28}$. Moreover let us put ${{\tilde{c}}}_3=2$ and ${{\tilde{c}}}_4=1$. With ${{\tilde{C}}}=10^{88}$ the assumptions of Lemma 12 are satisfied and we obtain

$$\begin{aligned} 2\exp (- 136.4)>|A'\log 2+B'\log 3+C'\log 5|>\frac{2}{p^{\alpha _1}q^{\beta _1}r^{\gamma _1}} \end{aligned}$$

which implies $\log s_1 \le 136.4$. Since the exact same computations also apply to (5) we also obtain that $\log s_2<136.4$ and therefore we have $M_0<136.4$ we conclude that $s_4<s_1s_2<\exp (272.8)$. Thus we have $M_4\le 272.8$.

**Step III** By a direct computation we are able to compute $B_p$, $B_q$ and $B_r$ by a brute force algorithm. The search implemented in Sage [14] takes on a usual PC a few seconds. In particular we obtain in the case that $S=\{2,3,5\}$ the bounds

$$\begin{aligned} \frac{B_p}{\log p}=18, \qquad \frac{B_q}{\log q}=13, \qquad \frac{B_r}{\log r}=8. \end{aligned}$$

By Proposition 2 this yields

$$\begin{aligned} \alpha _{\sigma (5)}\le 392, \qquad \beta _{\tau (5)}\le 247, \qquad \gamma _{\rho (5)}\le 169. \end{aligned}$$

In particular, we have $M_5=M_4\le 272.8$.

**Step IV** In this step we use the theorem of Bugeaud and Laurent (Lemma 5) to estimate the quantities ${\mathcal {C}}_p$ and obtain a new upper bound for $\alpha _{\sigma (6)}$ due to Proposition 3 (and the upper bounds for ${\mathcal {C}}_q$ and ${\mathcal {C}}_r$ by Propositions 5 and 6 in the “Appendix”). Since the computations for ${\mathcal {C}}_q$ and ${\mathcal {C}}_r$ are similar we give the details only for ${\mathcal {C}}_p$ for Case I note that the upper bounds for Cases II and III can be deduced similarly.

To find an upper bound for ${\mathcal {C}}_p$ we split up the set ${\mathcal {M}}_{x,y,z}$ into two subsets ${\mathcal {M}}'_{x,y,z},{\mathcal {M}}''_{x,y,z}\subseteq {\mathcal {M}}_{x,y,z}$ with

$$\begin{aligned}&{\mathcal {M}}'_{x,y,z}=\left\{ (a_x,b_x,c_x,a_y,b_y,c_y,b_z,c_z)\in {\mathbb {N}}^8\, :\right. \\&0\le a_x\log p+b_x\log q+c_x\log r,a_y\log p+b_y\log q+c_y\log r \le M_4, \; \text {and}\\&a_y<a_x\; \text {and}\; \left. 0\le b_z\le \frac{M_5}{\log q},0\le c_z\le M\right\} \end{aligned}$$

and

$$\begin{aligned}&{\mathcal {M}}''_{x,y,z}=\left\{ (a_x,b_x,c_x,a_y,b_y,c_y,b_z,c_z)\in {\mathbb {N}}^8\, :\right. \\&0\le a_x\log p+b_x\log q+c_x\log r,a_y\log p+b_y\log q+c_y\log r \le M_4, \; \text {and}\\&a_y<a_x\; \text {and}\; \left. 0\le b_z\le M,0\le c_z\le \frac{M_5}{\log r}\right\} \end{aligned}$$

and also consider the two quantities

$$\begin{aligned} {\mathcal {C}}'_p=\max _{{\mathcal {M}}'_{x,y,z}}\left\{ \nu _p\left( \frac{q^{b_z}r^{c_z}\left( p^{a_y}q^{b_y}r^{c_y}-1\right) }{q^{b_y}r^{c_y}-p^{a_x-a_y}q^{b_x}r^{c_x}}-1\right) \right\} \end{aligned}$$

and

$$\begin{aligned} {\mathcal {C}}''_p=\max _{{\mathcal {M}}''_{x,y,z}}\left\{ \nu _p\left( \frac{q^{b_z}r^{c_z}\left( p^{a_y}q^{b_y}r^{c_y}-1\right) }{q^{b_y}r^{c_y}-p^{a_x-a_y}q^{b_x}r^{c_x}}-1\right) \right\} . \end{aligned}$$

Of course we have ${\mathcal {C}}_p=\max \{{\mathcal {C}}'_p,{\mathcal {C}}''_p\}$ and we have to find upper bounds for ${\mathcal {C}}'_p$ (see Step IV (a)) and ${\mathcal {C}}''_p$ (see Step IV (b)). Similarly we can define ${\mathcal {C}}'_q, {\mathcal {C}}''_q,{\mathcal {C}}'_r$ and ${\mathcal {C}}''_r$ which will yield bounds for ${\mathcal {C}}_q$ and ${\mathcal {C}}_r$ respectively.

**Step IV (a):** In this step we compute an upper bound for ${\mathcal {C}}'_p$. We put

$$\begin{aligned} \eta _1=r,\qquad \eta _2=\frac{q^{b_z}(p^{a_y}q^{b_y}r^{c_y}-1)}{q^{b_y}r^{c_y}-p^{a_x-a_y}q^{b_x}r^{c_x}} \end{aligned}$$

and $b_1=c_z$ and $b_2=1$. We find that

$$\begin{aligned}&h(\eta _2) \le \max \{b_z\log q +\log (p^{a_y}q^{b_y}r^{c_y}-1),\\&\log (q^{b_y}r^{c_y}-p^{a_x-a_y}q^{b_x}r^{c_x})\} \le M_5+M_4 \end{aligned}$$

since we have $0\le b_z\le \frac{M_5}{\log q}$ and

$$\begin{aligned} 0\le a_x\log p+b_x\log q+c_x\log r,a_y\log p+b_y\log q+c_y\log r \le M_4. \end{aligned}$$

Applying Lemma 5 with this data we obtain a new hopefully smaller upper bound for $A=\alpha _{\sigma (6)}$.

**Step IV (b):** In this step we compute an upper bound for ${\mathcal {C}}''_p$. In this case we put $\eta _1=q$,

$$\begin{aligned} \eta _2=\frac{r^{c_z}(p^{a_y}q^{b_y}r^{c_y}-1)}{q^{b_y}r^{c_y}-p^{a_x-a_y}q^{b_x}r^{c_x}} \end{aligned}$$

and $b_1=b_z$ and $b_2=1$. We find that

$$\begin{aligned} h(\eta _2)\le \max \{c_z\log r +\log (s_y-1),\log (s_y-s_x)\}\le M_5+M_4 \end{aligned}$$

since we have $0\le c_z\le \frac{M_5}{\log r}$ and

$$\begin{aligned} 0\le a_x\log p+b_x\log q+c_x\log r,a_y\log p+b_y\log q+c_y\log r \le M_4. \end{aligned}$$

Again we apply Lemma 5 with this data and we obtain an upper bound for $A=\alpha _{\sigma (6)}$ in this case.

Similarly we find upper bounds in Cases II and III. We also find upper bounds for $B=\beta _{\tau (6)}$ and $C=\gamma _{\rho (6)}$ by using Propositions 5 and 6 instead of Propositions 3.

In the case that $S=\{2,3,5\}$ we obtain

$$\begin{aligned} A<7.17 \cdot 10^8,\qquad B<1.73\cdot 10^8, \qquad C<6.33\cdot 10^7. \end{aligned}$$

**Step V** We repeat the process of the Steps II–IV iteratively with the new found bounds for $M_0$ and *M* four more times and will find significantly smaller bounds. In the case that $S=\{2,3,5\}$ we obtain after five LLL-reductions the upper bounds $M_0<33.624$, $M_4=M_5<67.248$ and

$$\begin{aligned} A<4.51 \cdot 10^6,\qquad B< 1.3 \cdot 10^6,\qquad C< 1.01 \cdot 10^6. \end{aligned}$$

We implemented the Steps I–V in Sage. It took a usual desktop PC only a few seconds to obtain the bounds stated in Step V. Before we proceed with Step VI let us summarize what we proved so far in the case that $S=\{2,3,5\}$:

### Proposition 4

Assume that (*a*, *b*, *c*, *d*) is a $\{2,3,5\}$-Diophantine quadruple with $a<b<c<d$. Then $\log s_1,\log s_2 <33.624$ and $\log s_4<67.25$.

**Step VI** In this step we find all $\{p,q,r\}$-Diophantine triples (*a*, *b*, *c*) such that $\log (ab+1)=\log s_1,\log (ac+1)=\log s_2<M_0$ and $\log (bc+1)=\log s_4<M_4$. Therefore we proceed as follows:

1. We enumerate all *S*-units $s_4=p^{\alpha _4}q^{\beta _4}r^{\gamma _4}$ with $\log s_4<M_4$.
2. We enumerate all *S*-units $s_2=p^{\alpha _2}q^{\beta _2}r^{\gamma _2}$ with $\log s_2<\min \{\log s_4, M_0\}$ and $s_2\not \mid s_4$. In particular, we consider only those $s_2$ which satisfy $\gamma _2>\gamma _4$ in case that $\alpha _2\le \alpha _4$ and $\beta _2\le \beta _4$.
3. We compute for all pairs $s_2,s_4$ the quantity $B_c=\frac{s_4-s_2}{\gcd (s_2,s_4)}$. Due to Lemma 2 we have $c\le B_c$. If $(B_c-1)B_c+1<s_4$ we discard the pair $s_2,s_4$ since otherwise we would have
  $$\begin{aligned} bc+1<(B_c-1)B_c+1<s_4=bc+1, \end{aligned}$$
  a contradiction.
4. We enumerate all *S*-units $s_1=p^{\alpha _1}q^{\beta _1}r^{\gamma _1}$ with $s_1<s_2$ such that
  if $p=2$ and $\alpha _4,\alpha _2>1$, then $\alpha _1\le 1$;
  if $p\equiv 3\pmod 4$ and $\alpha _4,\alpha _2>0$, then $\alpha _1=0$;
  if $q\equiv 3\pmod 4$ and $\beta _4,\beta _2>0$, then $\beta _1=0$;
  if $r\equiv 3\pmod 4$ and $\gamma _4,\gamma _2>0$, then $\gamma _1=0$.
  That is we enumerate all *S*-units $s_1$ such that the content of Lemma 3 is not violated for the *S*-Diophantine triple (*a*, *b*, *c*).
5. For all these triples $(s_1,s_2,s_4)$ we compute $a_\square =\frac{(s_1-1)(s_2-1)}{s_4-1}$. We discard all triples $(s_1,s_2,s_4)$ for which $a_\square$ is not the square of an integer. For the remaining triples we compute $a=\sqrt{a_\square }$.
6. We discard all triples $(s_1,s_2,s_4)$ for which $b=\frac{s_1-1}{a}$ and $c=\frac{s_2-1}{a}$ are not integers.
7. We store all triples (*a*, *b*, *c*) in a list Triples.

In the case that $S=\{2,3,5\}$ the list Triples consists of the *S*-Diophantine triples

$$\begin{aligned}&\{(1, 3, 5), (1, 7, 9), (1, 15, 17), (1, 23, 89), (1, 5, 19), (1, 2, 4), (1, 5, 7), (1, 47, 49),\\&(1, 19, 485), (1, 7, 23), (1, 17, 19), (1, 31, 47), (1, 49, 119), (1, 7, 3749), (1, 3, 53),\\&(1, 11, 29), (1, 9, 71), (1, 2, 7), (1, 7, 17), (1, 4, 11), (1, 11, 49), (3, 13, 83),\\&(1, 17, 127), (2, 7, 1562), (1, 19, 1151), (1, 3, 8), (1, 9, 11), (1, 17, 47), (1, 159, 161),\\&(1, 29, 31), (1, 19, 71), (1, 127, 287), (1, 7, 107), (1, 89, 8191), (1, 24, 26),\\&(1, 44, 71), (1, 29, 431)\}. \end{aligned}$$

Let us note that Step VI is the bottleneck of our algorithm. It took more than six and a half hours to find all these triples using a usual PC (Intel i7-8700) on a single core.

**Step VII** Let us fix one *S*-Diophantine triple (*a*, *b*, *c*) from the list Triples computed in Step VI. Let us assume that this triple can be extended to a *S*-Diophantine quadruple. In this step we reduce the previously found huge upper bound for $s_3=ad+1$ by using the LLL-reduction (Lemma 12). Therefore we consider the inequality

$$\begin{aligned} \frac{b}{a}\cdot \frac{ad+1}{bd+1}-1=\frac{b-a}{abd+a}<\frac{1}{ad+1} \end{aligned}$$

and taking logarithms we obtain

10 $$\begin{aligned} \left| \log (b/a)+(\alpha _3-\alpha _5)\log p+(\beta _3-\beta _5)\log q+(\gamma _3-\gamma _5)\log r\right| <\frac{2}{s_3} \end{aligned}$$

We apply Lemma 12 to this Diophantine inequality that is we have

$$\begin{aligned} k=3,\quad \eta _1=\log r,\quad \eta _2=\log q,\quad \eta _4=\log p, \quad \eta _0=\log (b/a). \end{aligned}$$

Moreover we put $X_1=A$, $X_2=B$ and $X_3=C$, where *A*, *B* and *C* are the bounds for $\alpha _{\sigma (6)}, \beta _{\tau (6)}$ and $\gamma _{\rho (6)}$ found in Step V. We distinguish now between two cases.

**Case I** Let us assume that *p*, *q*, *r* and *a*/*b* are not multiplicatively dependent. For example this happens for the $\{2,3,5\}$-Diophantine triple (1, 7, 9). We compute $l({\mathcal {L}},y)$ by using Lemma 11 with

$$\begin{aligned} {\mathcal {A}}=\begin{pmatrix} 1 &{} 0 &{} 0 \\ 0 &{} 1 &{} 0 \\ \lfloor {C\log r}\rfloor &{} \lfloor {C\log q}\rfloor &{} \lfloor {C\log p}\rfloor \end{pmatrix}=\begin{pmatrix} 1 &{} 0 &{} 0 \\ 0 &{} 1 &{} 0 \\ \lfloor {{{\tilde{C}}}\log 5}\rfloor &{} \lfloor {{{\tilde{C}}}\log 3}\rfloor &{} \lfloor {{{\tilde{C}}}\log 2}\rfloor \end{pmatrix} \end{aligned}$$

and

$$\begin{aligned} y=(0,0,-\lfloor - {{\tilde{C}}} \log (b/a) \rfloor )^T=(0,0,-\lfloor - {{\tilde{C}}} \log 7 \rfloor )^T. \end{aligned}$$

With ${{\tilde{C}}}=10^{25}$ we obtain by Lemma 12 the bound $\log s_3<46.6$ in the case of the $\{2,3,5\}$-Diophantine triple (1, 7, 9).

**Case II** Let us assume that *p*, *q*, *r* and *a*/*b* are multiplicatively dependent and that $b/a=p^{x_p}q^{x_q}r^{x_r}$. Then Inequality (10) turns into

$$\begin{aligned} \left| (\alpha _3-\alpha _5+x_p)\log p+(\beta _3-\beta _5+x_q)\log q+(\gamma _3-\gamma _5+x_r)\log r\right| <\frac{2}{s_3}. \end{aligned}$$

Thus we compute $l({\mathcal {L}},y)$ by using Lemma 11 with

$$\begin{aligned} {\mathcal {A}}=\begin{pmatrix} 1 &{} 0 &{} 0 \\ 0 &{} 1 &{} 0 \\ \lfloor {C\log r}\rfloor &{} \lfloor {C\log q}\rfloor &{} \lfloor {C\log p}\rfloor \end{pmatrix} \end{aligned}$$

and $y=(0,0,0)^T$. We put $X_1=A+|x_p|$, $X_2= B+|x_q|$ and $X_3= C+|x_r|$ and ${{\tilde{C}}}$ sufficiently large and obtain an upper bound for $\log s_3$. For example in the case of the $\{2,3,5\}$-Diophantine triple (1, 3, 5) we have $x_2=x_5=0$ and $x_3=1$ and obtain with this strategy $\log s_3<39.4$ if we choose ${{\tilde{C}}}=10^{25}$.

Let us note that in the case that $S=\{2,3,5\}$ we obtain in all cases the upper bound $\log s_3<40$.

**Step VIII** Let $M_3$ denote the upper bound for $\log s_3$ found in Step VII. We enumerate all $ad+1=s_3=p^{\alpha _3}q^{\beta _3}r^{\gamma _3}$ with $\log s_3<M_3$ and for each triple (*a*, *b*, *c*) found in Step VI we compute $d=\frac{s_3-1}{a}$. We discard all quadruples (*a*, *b*, *c*, *d*) for which *d* is not an integer and for which $bd+1$ and $cd+1$ are not *S*-units.

Note that in the case that $S=\{2,3,5\}$ we have $M_3=40$. However a rather quick computer search yields that no triple can be extended to a quadruple and the proof of Theorem 1 is therefore complete.

### Remark 1

Finally let us note that the algorithm presented in this paper would also work for arbitrary *S* with $|S|\ge 3$ after some modifications. For instance in Step IV we would have to apply the results of Yu on linear forms in *p*-adic logarithms (e.g. the results from [15]) instead of the results due to Bugeaud and Laurent [1] on linear forms in two *p*-adic logarithms. This will lead to much larger bounds for $M_0$ and $M_5$ than what we get in the case $|S|=3$.

Moreover, Step VI in which we search for extendable triples seems to get unfeasible in the case $|S|\ge 4$. Thus all together a systematic search for *S*-Diophantine quadruples or even quintuples in the case $|S|\ge 4$ seems to be unfeasible with the method presented in this paper.

## Appendix: Bounds for $\beta _{\tau (6)}$ and $\gamma _{\rho (6)}$

In this appendix we explicitly state the upper bounds for $\beta _{\tau (6)}$ and $\gamma _{\rho (6)}$ we get by adjusting the proof of Proposition 3. A bound for $\beta _{\tau (6)}$ is given by:

### Proposition 5

Let us assume that $M> \frac{M_0+3M_5}{\log (\min \{p,q,r\})}$, $\tau (6)$ and *x* are complementary indices and that *y*, *z* is another pair of complementary indices, with $y\in \{1,2,4\}$. If $\beta _y=\beta _z<\beta _x$ (Case I) we put

$$\begin{aligned} {\mathcal {M}}_{x,y,z}= & {} \left\{ (a_x,b_x,c_x,a_y,b_y,c_y,a_z,c_z)\in {\mathbb {N}}^8\, :\right. \\&1\le p^{a_x}q^{b_x}r^{c_x}, p^{a_y}q^{b_y}r^{c_y}\le \exp (M_4),\; b_y<b_x,\; \text {and}\\&\left. \left( 0\le a_z\le \frac{M_5}{\log p},0\le c_z\le M \; \text {or}\;\; 0\le a_z\le M ,0\le c_z\le \frac{M_5}{\log r} \right) \right\} , \end{aligned}$$

if $\beta _x=\beta _z<\beta _y$ (Case II) we put

$$\begin{aligned} {\mathcal {M}}_{x,y,z}= & {} \left\{ (a_x,b_x,c_x,a_y,b_y,c_y,a_z,c_z)\in {\mathbb {N}}^8\, :\right. \\&1\le p^{a_x}q^{b_x}r^{c_x}, p^{a_y}q^{b_y}r^{c_y}\le \exp (M_4),\; b_x<b_y,\; \text {and}\\&\left. \left( 0\le a_z\le \frac{M_5}{\log p},0\le c_z\le M \; \text {or}\;\; 0\le a_z\le M ,0\le c_z\le \frac{M_5}{\log r} \right) \right\} , \end{aligned}$$

and if $\beta _x=\beta _y$ (Case III) we put

$$\begin{aligned} {\mathcal {M}}_{x,y,z}= & {} \left\{ (a_x,b_x,c_x,a_y,b_y,c_y,a_z,c_z)\in {\mathbb {N}}^8\, :\right. \\&1\le p^{a_x}q^{b_x}r^{c_x}, p^{a_y}q^{b_y}r^{c_y}\le \exp (M_4),\; b_x=b_y,\; \text {and}\\&\left. \left( 0\le a_z\le \frac{M_5}{\log p},0\le c_z\le M \; \text {or}\;\; 0\le a_z\le M ,0\le c_z\le \frac{M_5}{\log r} \right) \right\} . \end{aligned}$$

We define

$$\begin{aligned} {\mathcal {C}}_q&=\max _{{\mathcal {M}}_{x,y,z}}\left\{ \nu _q\left( \frac{p^{a_z}r^{c_z}\left( p^{a_y}q^{b_y}r^{c_y}-1\right) }{p^{a_y}r^{c_y}-p^{a_x}q^{b_x-b_y}r^{c_x}}-1\right) \right\}&\text {Case I},\\ {\mathcal {C}}_q&=\max _{{\mathcal {M}}_{x,y,z}}\left\{ \nu _q\left( \frac{p^{a_z}r^{c_z}\left( p^{a_y}q^{b_y}r^{c_y}-1\right) }{p^{a_y}q^{b_y-b_x}r^{c_y}-p^{a_x}r^{c_x}}-1\right) \right\}&\text {Case II},\\ {\mathcal {C}}_q&=\max _{{\mathcal {M}}_{x,y,z}}\left\{ \nu _q\left( \frac{p^{a_z}r^{c_z}\left( p^{a_y}q^{b_y}r^{c_y}-1\right) _q}{ \left( p^{a_y}r^{c_y}-p^{a_x}r^{c_x}\right) _q}-1 \right) \right\}&\text {Case III},\\ \end{aligned}$$

where $(\cdot )_q$ denotes the *q*-free part. Then

$$\begin{aligned} \beta _{\tau (6)}\le \left\{ \begin{array}{cl} \frac{M_4}{\log q}+{\mathcal {C}}_q &{} \text {Cases I and II;}\\ \\ \frac{M_4+B_q}{\log q}+{\mathcal {C}}_q &{} \text {Case III.}\end{array} \right. \end{aligned}$$

A bound for $\gamma _{\rho (6)}$ is given by:

### Proposition 6

Let us assume that $M> \frac{M_0+3M_5}{\log (\min \{p,q,r\})}$, $\rho (6)$ and *x* are complementary indices and that *y*, *z* is another pair of complementary indices, with $y\in \{1,2,4\}$. If $\gamma _y=\gamma _z<\gamma _x$ (Case I) we put

$$\begin{aligned} {\mathcal {M}}_{x,y,z}= & {} \left\{ (a_x,b_x,c_x,a_y,b_y,c_y,a_z,b_z)\in {\mathbb {N}}^8\, :\right. \\&1\le p^{a_x}q^{b_x}r^{c_x}, p^{a_y}q^{b_y}r^{c_y}\le \exp (M_4),\; c_y<c_x,\; \text {and}\\&\left. \left( 0\le a_z\le \frac{M_5}{\log p},0\le b_z\le M \; \text {or}\;\; 0\le a_z\le M ,0\le b_z\le \frac{M_5}{\log q} \right) \right\} , \end{aligned}$$

if $\alpha _x=\alpha _z<\alpha _y$ (Case II) we put

$$\begin{aligned} {\mathcal {M}}_{x,y,z}= & {} \left\{ (a_x,b_x,c_x,a_y,b_y,c_y,a_z,b_z)\in {\mathbb {N}}^8\, :\right. \\&1\le p^{a_x}q^{b_x}r^{c_x}, p^{a_y}q^{b_y}r^{c_y}\le \exp (M_4),\; c_x<c_y,\; \text {and}\\&\left. \left( 0\le a_z\le \frac{M_5}{\log p},0\le c_z\le M \; \text {or}\;\; 0\le a_z\le M ,0\le c_z\le \frac{M_5}{\log r} \right) \right\} , \end{aligned}$$

and if $\alpha _x=\alpha _y$ (Case III) we put

$$\begin{aligned} {\mathcal {M}}_{x,y,z}= & {} \left\{ (a_x,b_x,c_x,a_y,b_y,c_y,a_z,b_z)\in {\mathbb {N}}^8\, :\right. \\&1\le p^{a_x}q^{b_x}r^{c_x}, p^{a_y}q^{b_y}r^{c_y}\le \exp (M_4),\; c_x=c_y,\; \text {and}\\&\left. \left( 0\le a_z\le \frac{M_5}{\log p},0\le b_z\le M \; \text {or}\;\; 0\le a_z\le M ,0\le b_z\le \frac{M_5}{\log q} \right) \right\} . \end{aligned}$$

We define

$$\begin{aligned} {\mathcal {C}}_r&=\max _{{\mathcal {M}}_{x,y,z}}\left\{ \nu _r\left( \frac{p^{a_z}q^{b_z}\left( p^{a_y}q^{b_y}r^{c_y}-1\right) }{p^{a_y}q^{b_y}-p^{a_x}q^{b_x}r^{c_x-c_y}}-1\right) \right\}&\text {Case I},\\ {\mathcal {C}}_r&=\max _{{\mathcal {M}}_{x,y,z}}\left\{ \nu _r\left( \frac{p^{a_z}q^{b_z}\left( p^{a_y}q^{b_y}r^{c_y}-1\right) }{p^{a_y}q^{b_y}r^{c_y-c_x}-p^{a_x}q^{b_x}}-1\right) \right\}&\text {Case II},\\ {\mathcal {C}}_r&=\max _{{\mathcal {M}}_{x,y,z}}\left\{ \nu _r\left( \frac{p^{a_z}q^{b_z}\left( p^{a_y}q^{b_y}r^{c_y}-1\right) _r}{ \left( p^{a_y}q^{b_y}-p^{a_x}q^{b_x}\right) _r}-1 \right) \right\}&\text {Case III}, \end{aligned}$$

where $(\cdot )_r$ denotes the *r*-free part. Then

$$\begin{aligned} \gamma _{\rho (6)}\le \left\{ \begin{array}{cl} \frac{M_4}{\log r}+{\mathcal {C}}_r &{} \text {Cases I and II;}\\ \\ \frac{M_4+B_r}{\log r}+{\mathcal {C}}_r &{} \text {Case III.}\end{array} \right. \end{aligned}$$
